# A review of new emerging livestock-associated methicillin-resistant *Staphylococcus aureus* from pig farms

**DOI:** 10.14202/vetworld.2023.46-58

**Published:** 2023-01-10

**Authors:** Aswin Rafif Khairullah, Shendy Canadya Kurniawan, Mustofa Helmi Effendi, Sri Agus Sudjarwo, Sancaka Chasyer Ramandinianto, Agus Widodo, Katty Hendriana Priscilia Riwu, Otto Sahat Martua Silaen, Saifur Rehman

**Affiliations:** 1 Doctoral Program in Veterinary Science, Faculty of Veterinary Medicine, Universitas Airlangga. Jl. Dr. Ir. H. Soekarno, Kampus C Mulyorejo, Surabaya 60115, East Java, Indonesia; 2 Master Program of Animal Sciences, Department of Animal Sciences, Specialisation in Molecule, Cell and Organ Functioning, Wageningen University and Research, Wageningen 6708 PB, Netherlands; 3 Department of Veterinary Public Health, Faculty of Veterinary Medicine, Universitas Airlangga. Jl. Dr. Ir. H. Soekarno, Kampus C Mulyorejo, Surabaya 60115, East Java, Indonesia; 4 Department of Veterinary Pharmacology, Faculty of Veterinary Medicine, Universitas Airlangga. Jl. Dr. Ir. H. Soekarno, Kampus C Mulyorejo, Surabaya 60115, East Java, Indonesia; 5 Lingkar Satwa Animal Care Clinic, Jl. Sumatera No. 31L, Gubeng, Surabaya, Jawa Timur 60281, Indonesia; 6 Doctoral Program in Biomedical Science, Faculty of Medicine, Universitas Indonesia, Jl. Salemba Raya No. 6 Senen, Jakarta 10430, Indonesia

**Keywords:** livestock-associated-methicillin-resistant *Staphylococcus aureus*, pig, zoonosis, public health

## Abstract

Methicillin-resistant *Staphylococcus aureus* (MRSA) is a *S. aureus* strain resistant to β-lactam antibiotics and is often associated with livestock, known as livestock-associated (LA)-MRSA. Using molecular typing with multi-locus sequence typing, MRSA clones have been classified in pigs, including clonal complex 398. Livestock-associated-methicillin-resistant *S. aureus* was first discovered in pigs in the Netherlands in 2005. Since then, it has been widely detected in pigs in other countries. Livestock-associated-methicillin-resistant *S. aureus* can be transmitted from pigs to pigs, pigs to humans (zoonosis), and humans to humans. This transmission is enabled by several risk factors involved in the pig trade, including the use of antibiotics and zinc, the size and type of the herd, and the pig pen management system. Although LA-MRSA has little impact on the pigs’ health, it can be transmitted from pig to pig or from pig to human. This is a serious concern as people in direct contact with pigs are highly predisposed to acquiring LA-MRSA infection. The measures to control LA-MRSA spread in pig farms include conducting periodic LA-MRSA screening tests on pigs and avoiding certain antibiotics in pigs. This study aimed to review the emerging LA-MRSA strains in pig farms.

## Introduction

Methicillin-resistant *Staphylococcus aureus* (MRSA) is an *S. aureus* strain containing genes encoding *mec*A and *mec*C, which imparts resistance to β-lactam antibiotics [1]. In general, MRSA is divided into three types. The first type includes hospital-acquired (HA)-MRSA, which was first identified in humans in the late 1980s. The second one is community-acquired (CA)-MRSA), which was first discovered in humans in the mid-1990s [2], while the third includes livestock-associated (LA)-MRSA, which was first detected in humans in 2005 [3, 4].

Using molecular typing with multi-locus sequence typing, MRSA clones found in pigs have been classified as clonal complex 398 (CC398) with sequence type 398 (ST398) [5]. In pigs, LA-MRSA is becoming increasingly resistant to several antibiotics commonly used in livestock, such as tetracycline, aminoglycoside, trimethoprim, and penicillin [6, 7].

Most livestock animals are often infected with *S. aureus*. Recently, LA-MRSA has been identified in livestock used to produce animal-based foods, such as pigs [8, 9], cattle [10], goats [11], sheep [12], chicken [13, 14], and fish [15], and also in various food products, including pork [16], beef [17, 18], chicken meat [19–21], milk [22–25], dairy [26, 27], and fishery products [28]. The previous studies determined that pigs are one of the main reservoirs for several LA-MRSA strains [29, 30, 31], of which strain CC398 can be transmitted to humans [4, 32].

The transmission of LA-MRSA from pigs to humans is a major global concern, especially in countries with large-scale pig production, including European and American countries. Although several studies have shown that people working in the livestock industry are at increased risk of infection or colonization with LA-MRSA [33–35], LA-MRSA infection is also rising among the general population [36].

Microorganisms, including MRSA, are public health concerns as these strains or the genes encoding MRSA-related proteins can be transferred from pigs to humans [37]. The transmission of MRSA from pigs to humans is also closely associated with its transmission between pigs [37].

Usually, the number of LA-MRSA infection cases in humans is lower than the HA-MRSA and CA-MRSA cases. This might be because humans infected with LA-MRSA CC398 generally have different demographics as they are usually younger and have less severe clinical characteristics with shorter hospitalization periods [38]. Moreover, LA-MRSA CC398 infection is less severe than that with *S. aureus* [39], although LA-MRSA is a globally occurring *S. aureus* strain [40].

So far, despite a significant pig population in several countries, there is little information about the characteristics, distribution, and transmission of LA-MRSA in pigs [41]. Therefore, this review will explain the general meaning of LA-MRSA, its epidemiology, its transmission and risk factors in pigs, public health impact, and control in commercial pig farms.

## Livestock-associated Methicillin-Resistant *S. aureus*

Livestock-associated-methicillin-resistant *S. aureus* CC398 infection in pigs was first discovered and reported in several European countries, such as Denmark [33], Italy [42], Spain [43], Germany [44], and Portugal [45]. Then, it was reported in North America [46], South Africa [47], Australia [48], and Asia [49]. Initially, LA-MRSA CC398 was only associated with pigs. Subsequently, this strain was identified in calves [50] and poultry [51]. Livestock-associated-methicillin-resistant *S. aureus* CC398 has also been identified in turkeys [52] and dairy cattle [7]. So far, LA-MRSA CC398 infection is still rare in livestock [53]. The emergence of LA-MRSA in farm animals might be correlated with alternative and conventional livestock systems [54, 55], farm size [44, 56], and the use of mixed feed ingredients, such as zinc and disinfectants [57].

Livestock-associated-methicillin-resistant *S. aureus* spreads between farms through animal trade, such as piglets or calves sold by specialized producers [58]. Furthermore, meat products of livestock origin can be contaminated by LA-MRSA during processing. For example, approximately 2217 meat samples were examined in the Netherlands, and MRSA contamination was found in 10.7% pork, 6.2% lamb, 15.2% beef, 15.2% veal, and 35.3% turkey samples [59]. A study conducted in Germany showed 2.8% MRSA contamination in pork-based products [60]. In 2010, a 32% MRSA contamination rate was reported [61]. Similar MRSA contamination rates were also reported in the United States [62], Canada [63], and Taiwan [64].

Livestock-associated-methicillin-resistant *S. aureus* has not yet been associated with food poisoning as LA-MRSA isolates with clonal complex 398 (CC398) rarely carry the enterotoxin gene [65, 66]. However, this might change over time because LA-MRSA CC398 can re-acquire the immune evasion gene cluster (IEC), which is present during prophage and is a hallmark of *S. aureus* infection in humans. Certain IEC types carry the *sep* and *sea* genes [67]. In a previous study, 19% of LA-MRSA CC398 infections were reported in humans, of which only one isolate carried the *sea* gene [36]. Although LA-MRSA CC398 isolates carrying the *sep* and *sea* genes have not been found in pigs, CC398 isolates with the *sea* gene have been reported in horses [36]. The presence of LA-MRSA CC398 in cow milk indicates its colonization in the udder, which causes subclinical mastitis in dairy cows [68]. Meanwhile, LA-MRSA CC398 has also been identified in domestic [69] and industrial rabbits [70].

## Livestock-associated-Methicillin-Resistant *S. aureus* in Pigs

Livestock-associated-methicillin-resistant *S. aureus* was first identified in pigs in the Netherlands in 2005 [4]. This report showed that a pig farmer, a 6-month-old baby, and their parents living near a pig farm were infected with the same LA-MRSA CC398 strain. Immediately after this discovery, it was also reported in pigs in several other European countries, although with varying prevalence rates [4].

An extensive European survey detected LA-MRSA infections in pig farms in 17 out of the 26 analyzed countries. Higher LA-MRSA levels were associated with countries with higher pig farming densities [6]. Although the LA-MRSA strain in pigs is mainly the clonal complex 398 (CC 398), other clonal complexes have also been reported in pigs, including CC1, CC9, and CC97 [71].

Livestock-associated-methicillin-resistant *S. aureus* CC398 infections have also been reported in pigs in North America [72], while LA-MRSA CC9 infection has been shown in pigs in China [73] and Spain [74].

## Epidemiology LA-MRSA in Pigs

Since the first report on LA-MRSA in pigs in the Netherlands in 2005, it was subsequently reported in pigs in several other countries [4]. Numerous studies and surveys conducted in various countries have shown its prevalence rate and spread in pigs.

A comprehensive baseline study conducted in 2008 by the European Food Safety Authority [6] analyzed the prevalence rate of LA-MRSA in pig herds from European farms and found positive results in 12 out of 26 European countries. The average positive rate of LA-MRSA in various European countries is 14% and 26.9% in pig breeding and production farms, respectively. In addition to the basic studies, different European countries have also reported regional or national prevalence rates of MRSA in healthy pigs.

In an investigation conducted in Germany, the spread of LA-MRSA in pig production farms showed a positive prevalence rate ranging between 45% and 70% [44, 75, 76], which is higher than in other countries. Further, pig-fattening farms in Germany had more LA-MRSA-positive cases than pig-breeding farms. The number of LA-MRSA-positive pigs might be correlated with the pig density in each region.

Investigations in the Netherlands showed that the LA-MRSA positive prevalence rate in pig production farms was estimated to be 23%–71% [6, 34, 77, 78] and was particularly high in finishing farms. A significant increase in LA-MRSA prevalence was reported in pig herds between 2007 and 2008 in the Netherlands, which might be related to the transmission of LA-MRSA between different herds [77].

Further investigation indicated positive LA-MRSA infections in various European countries, including Denmark [33], Belgium [79], Portugal [80], and Croatia [81], with the prevalence rates varying from 16% to 100%. Apart from Europe, LA-MRSA has also been identified in swine production units in the United States [72, 82], Brazil [83, 84], Ecuador [85], and several Asian countries, including Japan [86], Korea [87], China [73, 88], Pakistan [89], Vietnam [90], and Thailand [91, 92].

A comparison of molecular typing results of LA-MRSA isolates in pigs showed regional differences in the distribution of genetic variants. In Europe, the United States, and Canada, the primary LA-MRSA strain found in pig production farms was CC398. The frequently found non-CC398 LA-MRSA strains include CC1, CC9, CC30, and CC97. The most common *spa* types found in Europe in the CC398 lineage were t011, t034, and t108, mainly identified in pig breeding and production farms [6].

The *spa* type t108 is very common in pigs in the Netherlands [76]. Meanwhile, in Italy, the *spa* type t899 was the dominant clone, accounting for 24% of all LA-MRSA isolates from pig production farms [6, 31]. Non-CC398 LA-MRSA strains, especially CC1 and CC97, are highly prevalent in Italian pig farms [93].

In Canada, a human MRSA clone has been identified in pig herds, namely Canadian methicillin-resistant *S. aureus*-2 (CMRSA-2), also known as USA100 [63]. Canadian methicillin-resistant *S. aureus*-2 accounts for 14%–15% of the LA-MRSA isolates in pigs in Canada. This strain is also the most common infectious HA-MRSA strain in Canada and is prevalent in humans in the United States [63]. Another strain, CMRSA-5 or USA500, was also isolated from pigs for the first time. This epidemic strain in humans is not common in Canada and has only been reported in horses [83, 84].

In Asia, non-CC398 LA-MRSA strains have been reported in pigs, while LA-MRSA CC9 strains have been mostly identified in pig farms in China [30, 88], Thailand [85, 92], Pakistan [89], and Vietnam [90]. The *spa* types associated with LA-MRSA CC9 are distributed with different geographic patterns. The *spa* type t4358 is the most common one found in pig farms in Spain [74], while *sp*a type t899 is predominant in pig farms in China [88] and Taiwan [94]. *Spa* type t337 carrying, the SCC*mec* type IX was found in pig farms in Thailand [95].

In most investigations, the researchers did not take sufficient samples from pigs to make statistical conclusions about the diversity and prevalence of LA-MRSA in pigs. However, investigations conducted in several countries have shown the emergence and spread of LA-MRSA in pigs worldwide (Table-1) [6, 32–44, 72, 79–81, 86–89, 92, 93].

**Table-1:** LA-MRSA prevalence of positive rate in various countries and years.

Country	Year	Prevalence/positive rate (%)	Detection method	Reference
Netherlands	2005	39%, n = 504	ORSAB, and PCR	[6]
Belgium	2007	90% n = 50	PCR	[79]
Denmark	2009	13%	PCR	[33]
Switzerland	2009	1.3%	PCR	[6]
German	2012	21%, n = 547	Agar diffusion, PCR	[44]
Croatia	2012	22%	PCR	[81]
USA	2013	4.6%, N = 1085	MRSA agar, PCR-sequencing	[72]
Italy	2014–2015	38%, n = 215	PCR	[93]
Netherlands	2015	81%, n = 558	High salt enrichment broth, PCR	[32]
Portugal	2016	41%, n = 75	Disk diffusion, PCR	[80]
Korea	2012–2016	7.9%, n = 1119	Disk diffusion, PCR	[87]
China	2016	49%, n = 899	MRSA agar, PCR	[88]
Japan	2017	3.1%, n = 420	PCR	[86]
Pakistan	2019	63%, n = 150	PCR	[89]
Thailand	2021	19.7%, n = 116	PCR	[92]

LA-MRSA=Livestock-associated-methicillin-resistant *Staphylococcus aureus*, PCR=Polymerase chain reaction, MRSA=Methicillin-resistant *Staphylococcus aureus*, ORSAB=Oxacillin resistance screening agar base

## Transmission LA-MRSA in Pigs

The transmission of LA-MRSA from pigs to humans is a major human health concern worldwide, especially in countries with large pig populations. Although studies have mostly shown an increased risk of LA-MRSA infection and colonization in people working in the swine industry [33–35], this rate has also risen in the general population [96, 97, 98].

## Pig–pig Transmission

Epidemiologically, LA-MRSA transmission between pigs is mainly responsible for its spread [99]. Since pigs are considered important reservoirs of LA-MRSA, studies have attempted to determine the transmission routes within pig farms and herds [99].

The transmission of LA-MRSA *through* direct contact is probably the main transmission route between pigs [37, 100]. Livestock-associated-methicillin-resistant *S. aureus*-positive pigs can transmit the infection to other LA-MRSA-negative pigs [101, 102]. Therefore, only LA-MRSA-positive pigs spread the infection in pig farms or even outside these farms *through* the purchase of LA-MRSA-positive pigs [78, 103]. In addition, several studies have shown LA-MRSA transmission between pigs in abattoirs due to the high density of livestock in cages [9, 53, 102]. Karlsen *et al*. [102] reported that LA-MRSA-negative pigs might rapidly become LA-MRSA-positive during transport to the abattoir and at the time of stunning, in addition to the transmission that occurs *through* indirect contact.

Livestock-associated-methicillin-resistant *S. aureus* can also be transmitted from sows to piglets [100, 104–106]. An experimental study demonstrated the transmission of LA-MRSA from sows to all newborn piglets [107]. Moreover, other studies also reported that piglets from LA-MRSA-positive sows tended to become LA-MRSA-positive [105, 106]. However, LA-MRSA was also seen in piglets from LA-MRSA-negative sows, indicating the involvement of other factors. In this context, Rosen *et al*. [106] reported differences in LA-MRSA colonization between different farrow-to-finish pig farms, that is, farms with high LA-MRSA colonization to those with low LA-MRSA colonization. In this study, each pig farm can be considered a closed system where different factors, such as environmental pollution, may affect the LA-MRSA colonization in pigs. Moreover, piglets can also become LA-MRSA carriers or might re-colonize LA-MRSA over time. Therefore, the colonization status of LA-MRSA in sows is important and must be considered while executing control measures. However, the differences in LA-MRSA prevalence between farms complicate the standardization of hygiene methods and require well-implemented control measures on each farm.

### Pigs–human transmission

Several studies have reported that people that work or live in pig farms, including farmers and their families, abattoir staff, and veterinarians, are at high risk of contracting LA-MRSA from pigs [4, 13, 33, 34, 43, 78, 108–112]. In Belgium, 37.8% of people working or living in 25 out of the 49 pig farms investigated were infected with LA-MRSA CC398 [108]. Another study reported that 9.5% of veterinarians in Belgium who participated in this investigation were LA-MRSA-positive, of which 7.5% were identified as LA-MRSA CC398 [112].

Normanno *et al*. [110] reported that 5.6% of registered abattoir staff were LA-MRSA-positive, which was significantly higher than the general prevalence rate in the Netherlands (0.1%) [113]. Although the exact transmission route between pigs and humans has not been fully ascertained, it is likely to be the same as the pig-to-pig transmission route. Livestock-associated-methicillin-resistant *S. aureus* can be transmitted *through* direct contact and indirectly through contaminated air or the environment. The presence of LA-MRSA in humans is mainly related to the frequency of contact with pigs and the prevalence of LA-MRSA in the pig herd [114, 115].

Krukowski *et al*. [116] reported that the LA-MRSA prevalence rate in young cattle farmers and their family members decreased from 26% to 11% without direct contact with cattle, with only 7% carrying LA-MRSA CC398. This indicates that LA-MRSA is a persistent and potentially harmful agent to humans. Moreover, Mascaro *et al*. [117] reported that after brief, direct contact with LA-MRSA-positive pigs, 17% of farmers initially detected LA-MRSA within 24 h, and 94% of breeders tested positive for LA-MRSA. Further, the LA-MRSA-positive individuals tested negative for LA-MRSA, while other LA-MRSA-positive breeders became LA-MRSA negative shortly after that. However, Zomer *et al*. [118] reported that approximately 59% of farmers who previously tested LA-MRSA-positive did not turn LA-MRSA-negative during the summer leave, indicating the absence of direct contact with pigs during summer leave did not affect LA-MRSA colonization in pig farmers. Therefore, further research is needed to determine the capacity of LA-MRSA CC398 to infect humans repeatedly.

### Transmission between humans

To date, LA-MRSA has rarely been reported outside communities exposed to LA-MRSA-positive pigs [35, 43, 98, 119–121]. Several studies analyzed the human-to-human transmission of LA-MRSA CC398 in hospitals. Based on observational data, Würtz *et al*. [122] reported that the relative nosocomial transmission risk for LA-MRSA CC398 was 0.28 compared with non-CC398 LA-MRSA genotypes. Another study showed that LA-MRSA CC398 was six times less infectious than non-CC398 LA-MRSA genotypes in Dutch hospitals [123]. Moreover, a recent genome sequencing analysis indicated that the LA-MRSA CC398 lineage originated in humans and then spread to livestock, such as pigs. This human-to-pig transmission was followed by a decrease in the transmission capacity, colonization, and virulence of MRSA in humans [124]. However, the risk of LA-MRSA transmission outside hospital care and healthy communities still needs to be analyzed.

Meanwhile, although LA-MRSA CC398 in pigs may not be highly contagious in humans, reports have shown skin infections associated with LA-MRSA CC398 infection [125] and more severe infections, such as abscess, cellulitis, necrotizing fasciitis [126, 127], endocarditis [128], and bacteremia [129, 130]. Mama *et al*. [131] could not find LA-MRSA CC398 in humans, but they reported a low prevalence rate of LA-methicillin-sensitive *S. aureus* (MSSA) CC398 in humans. Thus, although LA-MRSA CC398 infection is rare in humans, the public should remain vigilant and cautious.

Despite the increasing prevalence of LA-MRSA among humans, there are only a few cases of in-hospital illness caused by LA-MRSA [126, 132–136]. In a hospital in Kuwait, Boswihi *et al*. [133] reported that approximately 13% of LA-MRSA-positive patients who could not be typed by pulsed-field gel electrophoresis (PFGE) had an active infection compared with 42% of patients with LA-MRSA who could be typed by PFGE, which was less virulent than LA-MRSA. This is consistent with the fact that several virulence determinants have been identified among LA-MRSA CC398 [65, 129, 136–144]. However, it is necessary to continuously monitor the determinants and epidemiology of their virulence because several LA-MRSA CC398 isolates have been reported with important human virulence factors, including staphylococcal enterotoxins [65, 139] and bicomponent Panton-Valentine leukocidin [129, 137, 138, 141, 142]. In addition, pig-associated LA-MRSA CC398 was linked with increased MRSA infections in Northern Europe [98].

## Risk Factors LA-MRSA in Pigs

Theoretically, LA-MRSA could arise in pig herds due to the transfer of the genes encoding the *mec*A and *mec*C genes from coagulase-negative *Staphylococcus* and LA-MSSA [145]. However, as this is a rare occurrence, the transmission of LA-MRSA in pigs is assumed to occur due to several external risk factors in most cases.

### Purchase of pigs

Several studies have investigated the effect of swine trafficking on LA-MRSA transmission, indicating the presence of identical LA-MRSA strains or clones in supplying and receiving pig farms [78, 103, 146]. In one study, 79% of pig herds that received LA-MRSA-positive pigs from a supplier tested positive for LA-MRSA, whereas only 23% of pigs with LA-MRSA-negative pig suppliers tested positive for LA-MRSA [58]. Purchasing pigs from more than two pig suppliers was also associated with higher positive LA-MRSA levels [147]. However, two-network modeling studies of the Danish pig trade focusing on LA-MRSA concluded that the pig trade alone could not explain the rapid spread of LA-MRSA in the Danish pig population [148]. In an investigation conducted in Norway, 32 out of 51 pig farms that received pigs from farms that tested positive for LA-MRSA were still LA-MRSA negative, so purchasing pigs from LA-MRSA-positive pig farms might not necessarily result in LA-MRSA contamination in the receiving pig farms [146]. This is because some farms buy very few pigs or quickly change suppliers after disinfecting and washing the pig farms. In another case, LA-MRSA was reported on a supply hog farm. Thus, the pigs shipped are not necessarily LA-MRSA positive [146].

The number of imported pigs at the country level has been identified as a risk factor for s LA-MRSA [149]. However, it still has limited relevance with respect to the spread of LA-MRSA between countries [150].

### Use of antimicrobials and zinc

Livestock-associated-methicillin-resistant *S. aureus* isolates harbor genes encoding *mec*A and *mec*C that impart resistance to methicillin and other β-lactam antibiotics, such as cephalosporins, penicillins, and carbapenems [151]. In addition, most LA-MRSA is also resistant to tetracycline [33], which is often used in pig farming [33]. However, *S. aureus* is known to rapidly acquire resistance after exposure to several antibiotics [2], such as lincosamides, aminoglycosides, streptogramins, fluoroquinolones, phenicols, and macrolides [5, 152].

Inappropriate and excessive use of antibiotics triggers the emergence of LA-MRSA clones in pig farms [152]. Studies have evaluated the use of antibiotics as a risk factor in triggering the emergence and spread of LA-MRSA in pig farms [78, 147, 153]. However, antibiotic treatment had no significant effect on the LA-MRSA-positive pigs [58, 77]. The effect of antibiotic use is supported by many studies on LA-MRSA intervention [154, 155] and transmission [37, 107].

In most European countries, zinc is frequently used to treat diarrhea in weaning pigs. Piglets receive prescribed zinc supplementation at doses up to 2500 mg/kg of feed during the first 2 weeks after weaning [156]. Total zinc consumption for medical purposes was over 400 tons in 2015 [156]. This treatment affected the LA-MRSA-positive pigs, indicating a possible genetic link between *mec*A and *czr*C encoding for zinc resistance [157–159]. However, LA-MRSA clones carrying SCC*mec* type IV do not have this gene. Thus, not all LA-MRSA isolates are zinc-resistant [158].

However, in one study, Moreno-Flores *et al*. [107] observed an increase in LA-MRSA colonization in the noses of pigs fed with a zinc-supplemented diet [160]. In another study, zinc concentration in the feed was found to affect the colonization status of LA-MRSA in pigs [57]. A randomized controlled trial proved that using high concentrations of zinc (3000 mg/kg) increases the persistence and prevalence of LA-MRSA colonization in weaning piglets [161]. Thus, state policies have mandated that the use of zinc in therapeutic concentrations would be phased out by 2022 because of the potential risk associated with zinc supplementation in pig feed for the colonization and spread of LA-MRSA [161].

### Herd size and herd type

Several studies have identified that the herd type and size are risk factors for LA-MRSA transmission in pig farms [44, 77, 147, 153]. The risk of LA-MRSA transmission was found to be lower in the newborn piglets group compared to the wean-to-finisher or grower-to-finisher group due to the low number of new sows and the infrequent purchase of sows [44]. Organic pig farming might reduce this transmission risk [55].

The size of a large herd of pigs has been identified as a risk factor for LA-MRSA transmission [162]. Large pig herds usually have a higher turnover of pigs, resulting in more external contact with susceptible individuals [162]. Management practices in these pig farms might differ from those of smaller pig farms, with variations in the use of antibiotics, purchase of gilts, and hygiene measures based on the size of the herd [77].

### Management factors

A pig farm management system can also be a risk factor associated with the LA-MRSA-positive status in pig herds [147]. Disinfection of pig breeding pens before each new pig’s arrival was also related to the LA-MRSA-positive status in pigs [57]. However, this might be due to some LA-MRSA isolates carrying the gene encoding qacG, making them resistant to quaternary ammonium compounds [57, 163, 164]. In relation to a herd of LA-MRSA-positive pigs on a farm, several other risk factors, such as hog tooth cutting and vaccination of pigs, might also be associated with increased LA-MRSA colonization [154].

## Public Health Impact

Several studies have shown that LA-MRSA CC398 is highly transmissible and adaptable in pig populations during intensive farming. Moreover, as pigs act as reservoirs for LA-MRSA, there is widespread LA-MRSA contamination in pig farms [4]. Therefore, the control and eradication of LA-MRSA CC398 in pig farms with intensive pig farming procedures can become increasingly difficult. In addition, as LA-MRSA CC398 does not significantly impact the health of pigs, it is questionable if the implementation of an expensive national LA-MRSA control and eradication program is feasible. However, the ability of LA-MRSA CC398 to transfer from pigs to other pigs or from pigs to humans is a matter of serious concern because people who have frequent direct contact with pigs are at high risk of becoming infected with LA-MRSA CC398 [4, 13, 43, 108, 110], even though cases of LA-MRSA CC398 infection in humans are rare [120, 126, 131–136].

Livestock-associated-methicillin-resistant *S. aureus* CC398 in pigs has been found to have a lower nosocomial transmission [122, 123] and virulence rate than HA-MRSA clones [133, 134]. Considering this, LA-MRSA CC398 is considered an insignificant public health threat compared to the human-associated MRSA genotype. However, the ability of LA-MRSA CC398 to acquire resistance and virulence genes will potentially lead to the emergence of a more virulent strain. More importantly, pig-associated bacteria are reservoirs of resistance genes for the *cfr* gene that imparts resistance to five different classes of antibiotics, such as oxazolidinones, including linezolid, one of the few active antibiotics frequently used to treat MRSA infection in humans. They can also transfer the *vga* gene, which has an active role in developing resistance to pleuromutilins used in treating MRSA infection in humans and animals [152]. The transfer of these genes into HA-MRSA and CA-MRSA clones can be a serious public health problem, resulting in treatment failure [165]. Therefore, as the LA-MRSA control and eradication program can potentially fail, further investigations are needed to analyze the evolution of the LA-MRSA resistance and virulence genes associated with pigs.

## Controlling LA-MRSA in Pigs

The spread of LA-MRSA can be controlled in pig farms by periodically conducting LA-MRSA screening tests on pigs and avoiding certain antibiotics [166]. The government must sanction or warn farmers not to exceed the reasonable threshold for antibiotics to pigs [167]. For example, a policy was implemented in Denmark in 2010, where the government gave a “yellow card” to farmers who were caught using excessive antibiotics for pigs [168]. In 2016, the “yellow card” policy was updated to consider including a different class of antibiotics to reduce the use of antibiotics that are important in human medicine and that lead to the development of antibiotic resistance [169]. Since 2017, quinolones, cephalosporins, and tetracyclines have been weighted by a factor of 1.5, while 3^rd^ and 4^th^ generation fluoroquinolones have been weighted by a factor of 1.

The swine industry has also banned using 3^rd^ and 4^th^ generation cephalosporins, as these antibiotics have been associated with much higher rates of LA-MRSA transmission in pig farms [154]. In addition, several European countries have executed a national target to reduce the use of antibiotics used to treat pigs by 10% between 2010 and 2013 [154].

There are several control measures to tackle the spread of LA-MRSA, such as making the farmers working in pig pens change their clothes regularly and wash their hands before leaving the pen. Farmers and veterinarians need to include initiatives to protect themselves from LA-MRSA infection. To reduce the rate of LA-MRSA infection in pig herds and the risk of LA-MRSA entering from pig herds, routine antibiotic treatment for pigs needs to be discontinued because antibiotic therapy should only be implemented by veterinarians while conducting examinations. Moreover, pig farmers and veterinary staff should have access to consultation services, and investigations should be made to ensure the proper use of antibiotics. Further, the use of vaccines as an alternative measure could be increased [166].

It is important to spread awareness among farmers to maintain the cleanliness of pig pens to avoid indirect LA-MRSA contamination from the air and the environment contaminated. Routine checks should be conducted periodically to evaluate the prevalence rate of LA-MRSA in pig farms. The transmission route of LA-MRSA should also be analyzed in-depth, and international efforts need to be made to promote strategies to reduce antibiotic resistance [170]. In 2018, several European countries implemented mandatory hygiene programs for handling pigs [171].

## Conclusion

Livestock-associated-methicillin-resistant *S. aureus* CC398 is a pathogenic strain frequently identified in pigs. Livestock-associated-methicillin-resistant *S. aureus* can be transmitted through direct or indirect contact. Breeders, family farmers, abattoir staff, and veterinarians are particularly at risk of becoming infected with LA-MRSA from pigs. Control measures need to be taken to minimize the spread of LA-MRSA by conducting periodic LA-MRSA screening tests in pigs, stopping the use of certain antibiotics in pigs, and maintaining the cleanliness of the pig pens.

## Authors’ Contributions

ARK: Conducted the study and drafted the manuscript. SCK, SCR, AW, KHPR, OSMS, and SR: Desigend the study and drafted the manuscript under the guidance of MHE. SAS and MHE: Revised the manuscript. All authors have read and approved the final manuscript.
